# miR-155 Inhibition Sensitizes CD4+ Th Cells for TREG Mediated Suppression

**DOI:** 10.1371/journal.pone.0007158

**Published:** 2009-09-24

**Authors:** Heiko F. Stahl, Tanja Fauti, Nina Ullrich, Tobias Bopp, Jan Kubach, Werner Rust, Paul Labhart, Vassili Alexiadis, Christian Becker, Mathias Hafner, Andreas Weith, Martin C. Lenter, Helmut Jonuleit, Edgar Schmitt, Detlev Mennerich

**Affiliations:** 1 Boehringer Ingelheim Pharma GmbH & Co. KG, Respiratory Diseases Research, Genomics Group, Biberach an der Riss, Germany; 2 Johannes Gutenberg University, Institute of Immunology, Mainz, Germany; 3 Johannes Gutenberg University, Department of Dermatology, Mainz, Germany; 4 Institute of Molecular Biology and Cell Culture Technology, University of Applied Sciences, Mannheim, Germany; 5 Genpathway Inc., San Diego, California, United States of America; New York University School of Medicine, United States of America

## Abstract

**Background:**

In humans and mice naturally occurring CD4^+^CD25^+^ regulatory T cells (nTregs) are a thymus-derived subset of T cells, crucial for the maintenance of peripheral tolerance by controlling not only potentially autoreactive T cells but virtually all cells of the adaptive and innate immune system. Recent work using Dicer-deficient mice irrevocably demonstrated the importance of miRNAs for nTreg cell-mediated tolerance.

**Principal Findings:**

DNA-Microarray analyses of human as well as murine conventional CD4^+^ Th cells and nTregs revealed a strong up-regulation of mature miR-155 (microRNA-155) upon activation in both populations. Studying miR-155 expression in FoxP3-deficient s*curfy* mice and performing FoxP3 ChIP-Seq experiments using activated human T lymphocytes, we show that the expression and maturation of miR-155 seem to be not necessarily regulated by FoxP3. In order to address the functional relevance of elevated miR-155 levels, we transfected miR-155 inhibitors or mature miR-155 RNAs into freshly-isolated human and mouse primary CD4^+^ Th cells and nTregs and investigated the resulting phenotype in nTreg suppression assays. Whereas miR-155 inhibition in conventional CD4^+^ Th cells strengthened nTreg cell-mediated suppression, overexpression of mature miR-155 rendered these cells unresponsive to nTreg cell-mediated suppression.

**Conclusion:**

Investigation of FoxP3 downstream targets, certainly of bound and regulated miRNAs revealed the associated function between the master regulator FoxP3 and miRNAs as regulators itself. miR-155 is shown to be crucially involved in nTreg cell mediated tolerance by regulating the susceptibility of conventional human as well as murine CD4^+^ Th cells to nTreg cell-mediated suppression.

## Introduction

T cell activation and homeostasis critically rely on the balance between activating and repressing signals which lead to a multitude of different signal transduction pathways ensuring the regulation of gene expression. However, promoter-based regulation of gene expression does not ultimately lead to proper translation and to the expression of a given protein. In addition to direct control of gene transcription, post-transcriptional modifications seem to be very important for T cell development, homeostasis and activation as well. As recently demonstrated by the nTreg cell-specific Dicer knock out mouse, microRNAs (miR) seem to be pivotal not only for the proper development of T cells, but ultimately for the suppressive function of naturally occurring CD4^+^CD25^+^FoxP3^+^ regulatory T cells (nTregs) [1]–[3]. Among miscellaneous miRNAs the miR-155 is encoded by a small phylogenetically-conserved region of the proto-oncogene BIC, which was first described as a common site of viral DNA integration in virally-induced lymphomas in chicken [4], [5]. Upon activation, miR-155 is expressed in several types of immune cells, including B- and T-cells [6], [7], macrophages and dendritic cells indicating its important role in the activation of these cells.

In 2007, crucial function of miR-155 in the immune system was proven by the miR-155−/− knock out mouse from Rodriguez et al. [8] respective Thai et al. [9]. These mice show a severe autoimmune phenotype in the lung which is characterized by leukocyte invasion in bronchoalveolar lavage fluids (BALF) and increased airway remodeling, suggesting that miR-155 plays a role in regulating the response of the immune system to self-antigens. However, little is known about the target genes that are regulated by miR-155. Recently, Lu et al. demonstrated that FoxP3-dependent regulation of miR-155 maintains the competitive fitness of murine nTregs cells by targeting SOCS1 [10].

In this context, nTregs are pivotal for preventing excessive or misguided immune responses especially against self-antigens by controlling not only potentially autoreactive T cells, but virtually all cells of the adaptive and innate immune system. Although the molecular mechanism underlying nTreg-mediated suppression seems to rely on the cAMP-mediated activation of Protein Kinase A in the responder cells [11], the activation status of the target cell is crucial in determining the outcome of this suppression [12].

To gain further insight into nTreg development and function, several DNA-microarray analyses with murine nTreg were performed. However, comparative analyses of human CD4^+^ T helper cells (Th) and human nTregs are sparse. We addressed this issue by profiling RNAs of resting and activated Th cells, as well as of nTregs isolating T cell populations from human leukapheresis products [13]. The RNAs of ten human healthy donors were hybridized to Affymetrix Human Exon Arrays. Among others, the microRNA155 precursor gene BIC was revealed to be strongly up-regulated upon activation in both human cell types. In addition, we performed duplicates of FoxP3 ChIP-Seq experiments (FoxP3 antibody-mediated chromatin immunoprecipitation followed by Illumina sequencing of DNA fragments) of human activated Th cells and nTregs. The analysis resulted in two dozen of FoxP3 bound/regulated miRNAs, whereas miR-155 is one of these.

To examine the role of miR-155 in nTreg cell-mediated suppression, we modulated the expression level of this miRNA in primary mouse and human CD4^+^ Th cells, as well as nTregs. While no significant effect in suppressive capacity could be seen in nTregs, the modulation of the expression level of miR-155 in CD4^+^ Th cells clearly demonstrated a crucial role for miR-155 as a ‘sensor’ for nTreg cell-mediated suppression: Increased miR-155 levels in both human and mouse, CD4^+^ Th cells led to a reduced susceptibility to nTreg cell-mediated suppression, whereas decreased miR-155-levels resulted in a more pronounced suppression by nTregs.

## Results and Discussion

**Figure 1:**
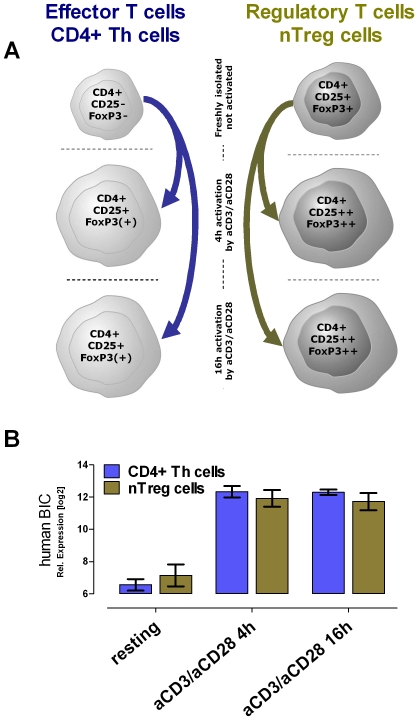
Human Exon Array Genechip expression profiling showed BIC as one of the highest up-regulated genes after T cell activation. (A) Schematic overview of different T cell populations out of 10 human donors. Expression profiles were analyzed of freshly isolated resting CD4^+^ Th cells and of nTregs. In addition, both populations were profiled upon 4 h and 16 h anti-CD3/anti-CD28 stimulation. (B) The expression profiling revealed the BIC transcript specifically up-regulated upon activation in both populations: the CD4^+^ Th cells and nTregs. The median relative expression level of BIC in logarithmic scale and the standard deviation is shown (n = 10).

Autoimmune diseases are characterized by hypersensitive immune responses to self-antigens. One proposed explanation for autoimmune pathogenesis is that naturally occurring CD4^+^CD25^+^ regulatory T cells (nTregs) lose their suppressive potential. Another possibility is that CD4^+^ Th cells have become insensitive to nTreg cell-mediated suppression. To identify the molecular mechanisms underlying the activation of T cells respective Treg cell-mediated suppression, we performed gene expression profiling of primary human T cells freshly-isolated by leukapheresis [14]. Using this method, we were able to isolate a sufficient number of T cells such that in vitro expansion was not necessary. Per donor, more than 5.0×10^7^ pure nTregs and more than 2.5×10^8^ pure CD4^+^ Th cells were obtained (Figure S1: FACS analysis of freshly isolated, not expanded, human CD4^+^ Th cell and nTreg cell populations). We compared, in a so-called “paired” analysis, resting versus 4 h and 16 h activated nTregs, to similarly activated CD4^+^ Th cells from the same healthy volunteer. We distinguished between early and late activated genes (Fig. 1A). To gain statistically relevant data, the study was performed with T cells from 10 different healthy human volunteers (n = 10).

We identified the proto-oncogene BIC that encodes the miRNA-155, among the genes most significantly up-regulated upon activation (Fig. 1B). In concordance, Cobb et al. [15] found miR-155 to be the most up-regulated microRNA of all analyzed miRNAs expressed in CD4^+^ Th cells activated for 3 days. These results, together with data from the miR-155 knockout mice, which exhibit an enhanced inflammation and onset of autoimmune diseases [8], [9], suggest an important role of miR-155 in T cell function.

### BIC is up-regulated in activated CD4^+^ Th and nTregs and is processed into mature miR-155

**Figure 2:**
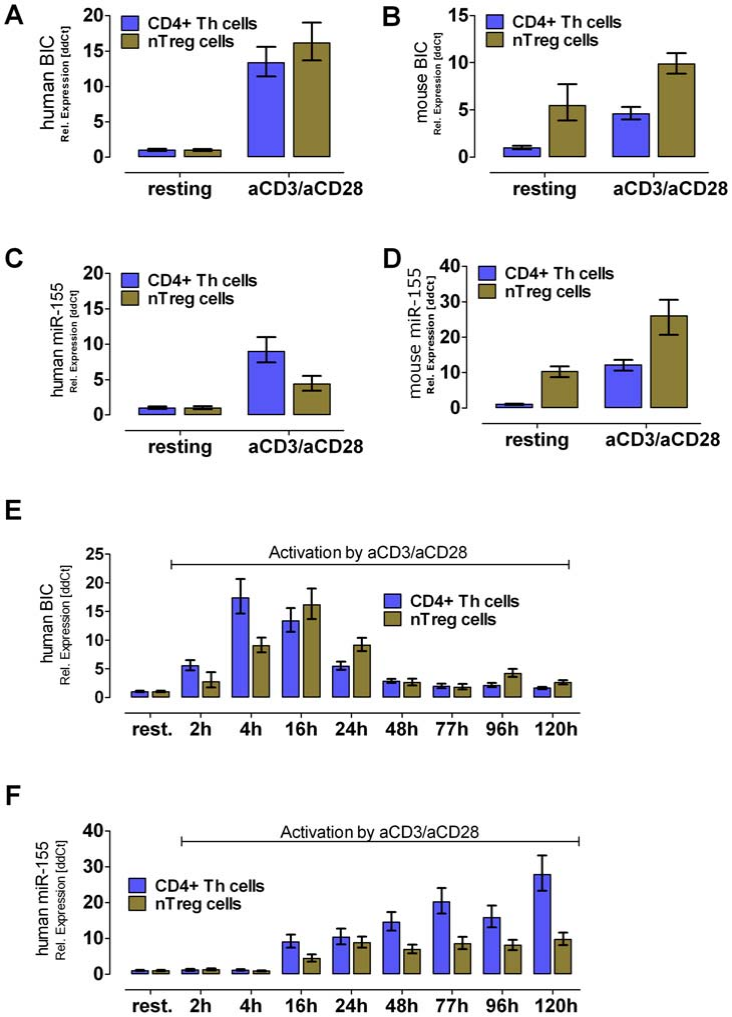
RT-PCR expression analysis of pre-mature BIC transcript and its processed microRNA miR-155 in mice and men. The BIC transcript is strongly up-regulated upon activation using anti-CD3/anti-CD28 mAb in human donors (A) and in C57/BL6 mice (B). Whereas, in C57/BL6 mice the BIC transcript as well as the matured miR-155 was found to be higher expressed in nTregs than in CD4^+^ Th cells (B) and (D) (n = 3). In human cells, BIC and the matured form miR-155 were not present in resting cells, but strongly elevated levels were found in Th cells as well as in nTreg cells after TCR activation (A) and (C) (n = 4). Analyzing the kinetic of BIC/miR-155 expression RNA was collected using an activation time course experiment. (E) The activation of human T cell populations showed a temporary activation of the BIC transcription. Whereas, the CD4^+^ Th cells reached their maximum after 4 h upon activation, the peak for nTreg cells is shifted to the 16 h time point. (F) The levels of matured miR-155 were found to permanently increase within time (until 120 h) in activated CD4^+^ Th cells, whereas in nTregs a plateau was reached after 24 h. All values were calculated as relative fold changes using the ddCT method. As normalizer RNA Pol II (human & mouse BIC) as well as U18 (human miR-155) and 5S (mouse miR-155) were used.

Validation of BIC expression was conducted by real time PCR (RT-PCR) using miRNA/RNA preparations from donors which were independent of the original 10 healthy human volunteers used for the array-based expression profiling. Real-time PCR analysis revealed a 14-fold up-regulation of human BIC upon activation compared resting CD4^+^ Th cells and resting nTregs (Fig. 2A). This tendency could be validated in mice as well (Fig. 2B). In accordance with results published by Haasch et al 2002 [6], our data show that BIC is hardly detectable in resting human T cells, but is strongly up-regulated rather early upon activation. To determine, if BIC is processed into mature microRNA, murine and human miR-155-specific cDNA was generated and analyzed by Taqman RT-PCR. As depicted in Fig. 2C (human) and Fig. 2D (mouse), BIC was processed into mature miR-155 in both species. Interestingly, resting murine nTregs showed an elevated basal level of the BIC transcript and of mature miR-155 when compared to human resting nTregs. This could be confirmed by the recently published paper of Lu et al. [10]. They also showed a 6-fold higher expression of miR-155 in mouse peripheral FoxP3 positive T cells compared to FoxP3 negative T cells. Assuming that miR-155 will be regulated in mice by the transcription factor FoxP3.

To analyze the kinetics of BIC transcription and subsequent miR-155 maturation upon activation, a time course experiment was performed using human T cells. BIC mRNA expression peaked after 4 h in human CD4^+^ Th cells, and after 16 h in Tregs (Fig. 2E). Thereafter, expression of BIC decreased most likely because it is processed to mature miR-155. In both populations, the expression of BIC returned to basal levels after 77 h. The same approach was used to determine the level of the mature miR-155. Whereas no detectable expression was seen in resting human CD4^+^ Th or nTreg cells, both cell types significantly expressed miR-155 upon 16 h of activation. In nTregs, the maximal expression level was reached after 24 h of activation and remained at this moderate plateau for the duration of the experiment. In CD4^+^ Th cells, the miR-155 level increased over time without reaching a plateau even after 120 h of activation (Fig. 2F). Based on this finding, one could argue that CD4^+^ Th cells need miR-155 for proper proliferation, whereas it plays an inferior role in peripheral human nTregs since they are anergic and do not proliferate *in vitro*. Our findings support the results of Lu et al., that miR-155 is involved in T cell proliferation and promoting the fitness of lymphocytes by targeting the IL2-signaling regulator protein SOCS1 [10].

### miR-155 expression is not necessarily regulated by FoxP3

**Figure 3:**
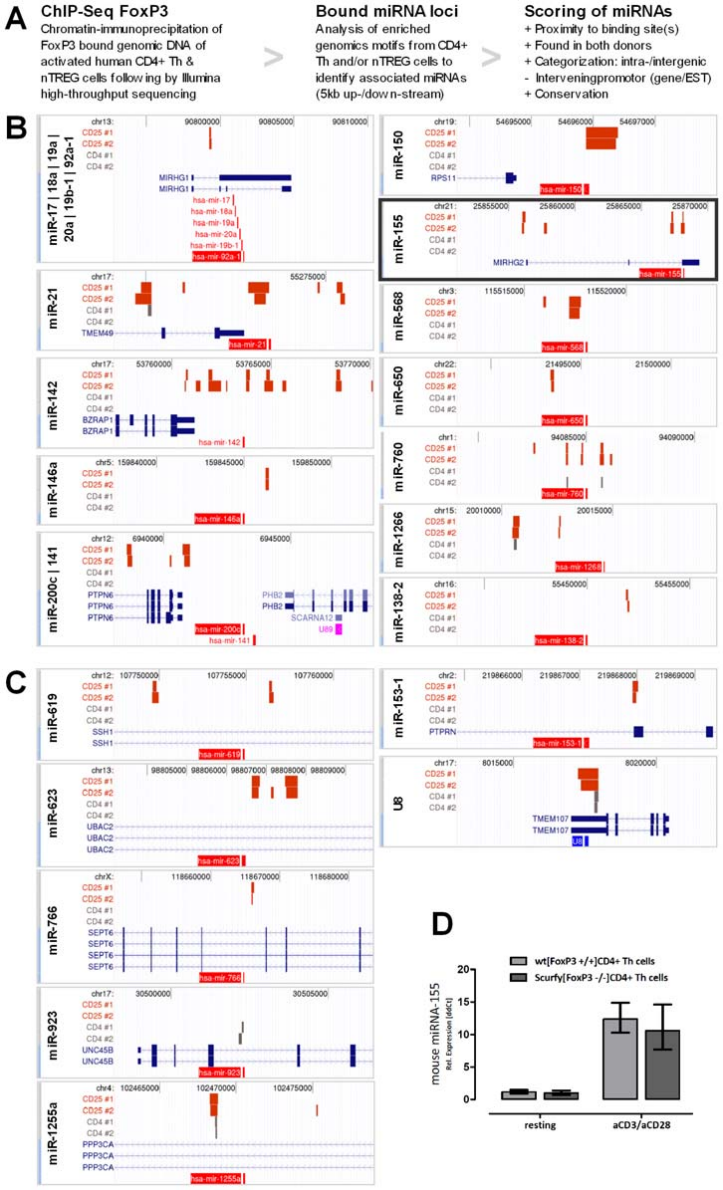
BIC/miR-155 expression is not necessarily regulated by FoxP3. (A) Schematic overview of the ChIP-Seq analysis workflow. Genomic loci of all significantly and reproducible FoxP3-bound micro-RNAs are shown: intragenic micro-RNA (B) and intergenic located micro-RNAs (C). Each genomic capture shows in light red the micro-RNA(s) and in blue the overlapping annotated gene(s) including the intro/exon structure(s). In dark red (CD25^+^/nTREG) and in gray (CD4^+^ Th cell) the FoxP3-bound genomic regions of both donors are symbolized. In addition, every capture contains the underlying chromosome including the basepair coordinates. The visualisations were generated using the UCSC genome browser (human genome assembly of March 2006). (D) Using Taqman RT-PCR, the expression analysis of T lymphocytes for mature miR-155 showed no significant difference between wild type C57/BL6 and FoxP3 mutated *Scurfy* mice. Post activation (19 h) an increased expression of matured miR-155 was detectable, in both CD4^+^ Th cells of wild type and *Scurfy* mice. All values were calculated as relative fold changes using the ddCT method. As normalizer 5S was used (n = 3).

In 2007, Zheng et al. [16] and Marson et al. [17] published mouse FoxP3 ChIP-on-CHIP analyses studies. They affirmed FoxP3 regulates the expression of BIC/miR-155 and that BIC/miR-155 is a direct target of FoxP3 in mice. To prove this hypothesis, we performed human FoxP3 ChIP-Seq experiments using activated (16 h) human CD4^+^ Th cells and nTregs of two independent donors (Fig. 3A). A miRNA-focussed bioinformatic analysis revealed at least 24 different miRNA loci, which are significantly and reproducibly detected to be bound by the transcription factor FoxP3. Due to their genomic localization 18 miRNAs were categorized as intergenic (Fig. 3B) und 6 miRNAs as intrageneic (Fig. 3C). A comprehensive list of FoxP3-bound micro-RNA-associated genomic loci can be found as a data file (table S1). The table also contains the miRNAs which are located nearby or directly within a promoter region of an annotated gene. The binding of FoxP3 to these regions can't be differentiated between specific gene and/or miRNA regulation. The group of intergenic FoxP3 bound miRNA targets (Fig. 3B) confirms the finding of Zheng et al. [16] and Marson et al. [17], that FoxP3 binds the genomic BIC/miR-155 locus in human regulatory T cells. Surprisingly, no binding of FoxP3 to BIC/miR-155 was detectable in CD4+ Th cells, which also express FoxP3 after activation.

To further understand the role of FoxP3 in the regulation of BIC/miR-155 expression, we compared the miR-155 levels of resting and activated CD4^+^ Th cells from FoxP3-mutant *scurfy* mice (*scurfy* mice lack the functional FoxP3 protein) to those of *wt* C57/Bl6 mice. CD4^+^ Th cells from *Scurfy* mice surprisingly revealed the same level of induction of miR-155 upon activation (Fig. 3D) as wild type CD4^+^ Th cells.

Therefore, we postulate that BIC/miR-155 expression and maturation of miR-155 seem to be not necessarily regulated by FoxP3 in CD4^+^ Th cells, even though human and mouse CD4^+^ Th cells expressed FoxP3 at moderate levels upon activation.

### miR-155 in CD4^+^ Th cells acts as a ‘sensor’ for nTreg cell-mediated suppression

**Figure 4:**
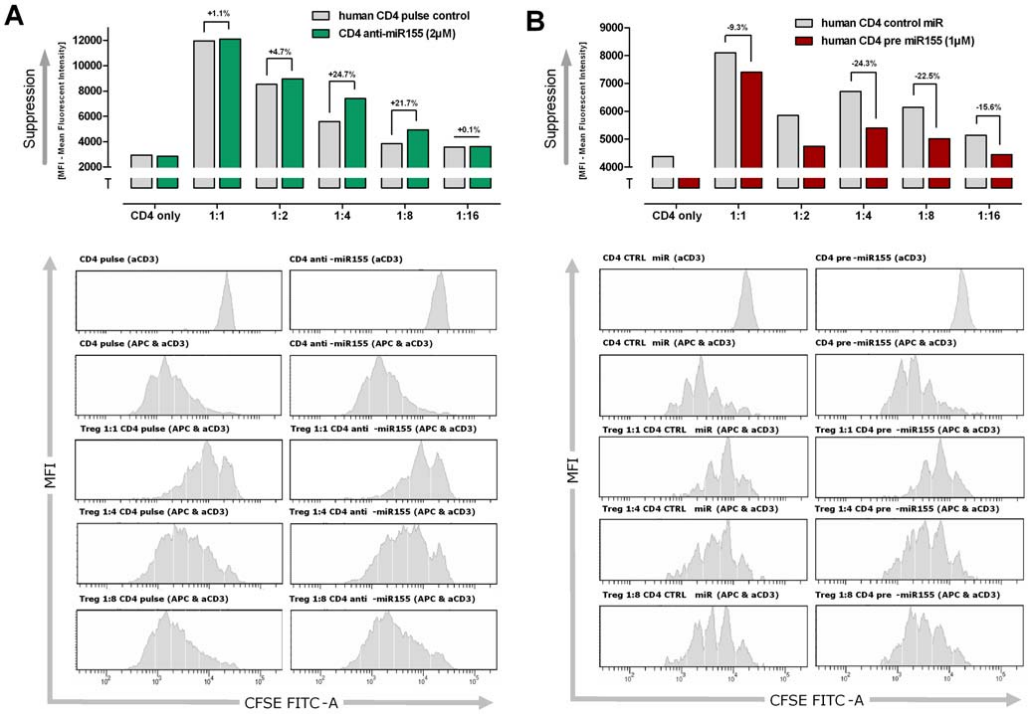
Modulation of miR-155 levels in CD4+ Th cells influenced the susceptibility to nTreg-mediated suppression. (A) Blocking the biological available miR-155 by transfection of synthetic anti-miR-155 molecules led to an increased sensibility for nTreg-mediated suppression. Depending on the ratio of CD4^+^ Th cells to nTregs (4∶1 and 8∶1) a nearly 25% increased susceptibility for suppression of proliferation was observed. The CD4^+^ Th cell population alone showed no change in proliferation rate in comparison to the pulsed only control population. (B) Increasing the miR-155 levels within CD4^+^ Th cells by transfection of synthetic miR-155 decrease the susceptibility for nTreg-mediated suppression measured in CFSE proliferation assays. Parallel assay setup revealed a nearly 25% decreased sensibility for suppression of proliferation. The CD4^+^ Th cell population alone showed also an elevated proliferation in comparison to the control-miRNA transfected population. Shown are the MFI (mean fluorescence intensity) of CFSE labelled CD4^+^ Th cells after four days of activation (aCD3 and APC). The bar plot diagrams are indicating the grade of suppression levels. The added numbers are showing the differences (%) for the susceptibility of nTreg-mediated suppression of proliferation of the activated CD4^+^ Th cells.

BIC is known as a proto-oncogene and its over-expression respective the subsequent up-regulation of miR-155 levels are associated with the development of B cell lymphoma [4], [5], [7]. The up-regulation of miR-155 is also observed in primary human and murine T cells from healthy donors as well. Therefore we addressed the functional consequence of miR-155 elevation on the proliferative capacity of CD4^+^ Th cells. We transfected human primary CD4^+^ Th cells with a human miR-155-specific inhibitor and analyzed the proliferative capacity of these cells in CFSE-based proliferation assays. Inhibition of miR-155 had no measurable effect on the proliferation of CD4^+^ Th cells upon polyclonal or allogeneic stimulation. In sharp contrast, miR-155 inhibitor-treated CD4^+^ Th cells showed a much higher susceptibility to nTreg-mediated suppression than control-transfected responder CD4^+^ Th cells (Fig. 4A). Whereas, modulation of miR-155 in nTregs cells did not alter their suppressive capacity (data not shown), as reported by Lu et al. [10].

**Figure 5:**
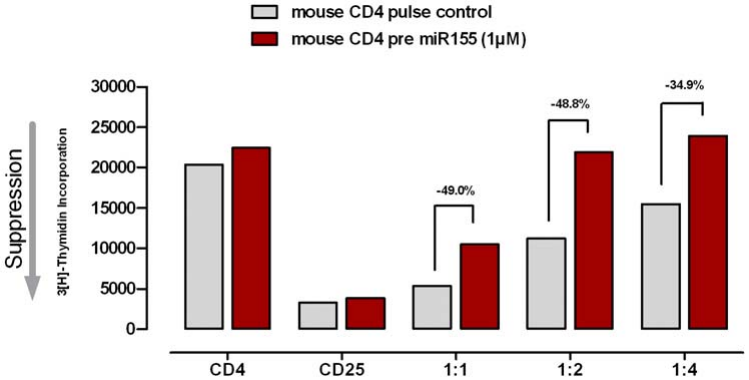
Overexpression of mature miR-155 rendered murine CD4^+^ Th cells unresponsive to nTreg-mediated suppression. Suppression assay performed with naïve murine CD4^+^ Th cells and murine nTregs: Th cells transfected with pre miR-155 showed up to 40% decreased susceptibility for nTreg cell-mediated suppression compared to the control-transfected CD4^+^ Th cells. Shown is the ^3^H-thymidine uptake after 18 h. The bar plot diagrams are indicating the grade of suppression levels. The added numbers are showing the differences (%) for the susceptibility of nTreg-mediated suppression of proliferation of the activated CD4^+^ Th cells. One representative experiment out of at least three experiments is shown.

To further corroborate these findings, human CD4^+^ Th cells were transfected with human mimic-miR-155 or its respective human pre-miR-155 (precursor) to analyze whether this evokes the opposite effect, namely rendering CD4^+^ Th cells unresponsive to nTreg cell-mediated suppression. To this end, miR-155, pre miR-155 as well as control-transfected CD4^+^ Th cells where co-cultured with nTregs at different ratios. Interestingly, overexpression of miR-155 resulted in a strongly decreased susceptibility of the CD4^+^ Th cells to nTreg cell-mediated suppression (Fig. 4B). Equivalent experiments were performed with naïve murine CD4^+^ Th cells and murine nTregs. Murine CD4^+^ Th cells transfected with murine pre-miR-155 showed an up to 40% decreased susceptibility to nTreg cell-mediated suppression compared to the control-transfected CD4^+^ Th cells, underscoring the results obtained with human T cells (Fig. 5). Again, increasing the levels of miR-155 in murine or human nTregs did not significantly influence their ability to suppress CD4^+^ Th cells (data not shown). Previous work has shown that a loss of miR-155 in Tregs did not impair their sensitivity to impair Treg cell suppressor function, whereas miR-155 is involved during thymic differentiation by promoting the fitness and the proliferative potential of differentiating nTregs [10].

### In silico analysis of putative miR-155 mRNA targets in CD4^+^ Th cells revealed 93 genes

For a detailed understanding on how miR-155 acts in CD4^+^ Th cells as a ‘sensory molecule’, it is necessary to know its target genes. Accordingly to their mechanism of action, miRNAs are crucial posttranscriptional regulators of gene expression by decreasing the abundance or translational efficiency of RNAs. Assuming that miR-155 regulates RNA-targets responsible for keeping CD4^+^ Th cells in a resting state, the potential candidates have to be negatively affected upon activation of CD4^+^ Th cells. RNA targets that are regulated in siRNA-like manner with final degradation of target mRNA are therefore detectable using DNA microarrays. Based on the integration of microRNA databases (miRanda, PicTar, TargetScanS) for in silico prediction [18] of miR-155 targets together with data obtained from the Affymetrix Exon Array profiling, we were able to generate a list of 93 putative miR-155 targets (table S2 & figure S3 for detailed description). For confirmation of the in silico prediction, we performed a detailed quantitative Taqman RT-PCR analysis for some genes to monitor their transcriptional down-regulation upon activation of miR-155 (figure S4). The best predicted miR-155 target with the highest seed match score is the transcription factor BTB and CNC homology 1 (Bach1, Figure S4A), which has been published recently to be regulated by miR-155 [19], [20].

Similar investigations addressed the role of miR-155 in overexpressing transgenic mice, which leads to pre-B cell proliferation in bone marrow and spleen, followed by high grade B cell neoplasms [21]. Although the precise role of miR-155 in promoting B cell lymphomagenesis is unclear, Dorsett et. al [22] studied the interaction miR-155/AID-RNA (activation-induced cytidine deaminase) after finding AID expression was 1.6 fold increased in miR-155-deficient B cells. In addition to its role in B cell proliferation, it was shown that miR-155 acts also as tumor suppressor by reducing potentially oncogenic translocations generated by AID. Vigorito et. al [23] identified the transcription factor Pu.1 to be regulated by miR-155 after profiling of miR-155-deficient B cells. Overexpression of Pu.1 impairs the emergence of IgG1-positive cells in vitro, its expression need to be down-regulated to permit class-switched B-cells to undergo differentiation to plasma cells [24], [25].

In contrast, in vitro analysis of T cell differentiation revealed no differences between miR-155-deficient T cells compared to wild-type cells. Nevertheless, it was shown the tendency of miR-155-deficient T cells to differentiate into Th2 cells [8], [9]. According to that phenotype the miR-155 target SOCS1 was indicated to be a regulator for balancing the Th1/Th2 cell differentiation [10] leading to a Th1 cell differentiation blockade by suppression of IL12 and IFN-γ signalling when miR-155 is deleted [26], [27]. It was shown, that miR-155 is required for nTreg cell homeostasis in the presence of limiting amounts of IL-2 because miR-155 is regulating the IL2 signalling repressor SOCS1 [10]. Confirming that phenotype also in CD4+ Th cells, we were able to show a strong up-regulation of IL-2 expression in murine CD4^+^ Th cells (13-fold) upon transfection of mimic-miR-155 (data not shown). To strengthen the thesis of miR-155 regulated IL2 signalling, we also affirmed a significant down-regulation of IL2 mRNA expression in miR-155 inhibitor transfected human CD4+ Th cells (data not shown).

### Conclusion

Foxp3 is essential for the normal development of nTREGs. In the absence of micro-RNAs, nTregs develop but fail to maintain immune homeostasis, leading to a scurfy-like disease. Recent work using Dicer-deficient mice irrevocably demonstrated the importance of miRNAs for the development of nTreg and for nTreg cell-mediated tolerance. Further investigation of FoxP3 downstream targets, certainly of bound and regulated miRNAs will reveal the associated function between the master regulator FoxP3 and miRNAs as regulators itself. We described in detail the transcriptional changes of miR-155 in primary human and murine T lymphocytes and that the expression and maturation of miR-155 seem to be not necessarily regulated by FoxP3 in CD4^+^ Th cells. In combination with miR-155 mediated elevation of IL-2 expression in CD4^+^ Th cells, we could demonstrate that raised miR-155 levels in human and murine CD4^+^ Th cells not only induces cell proliferation, but moreover renders CD4+ Th cells to become insensitive to nTreg cell-mediated suppression.

## Materials and Methods

### Preparation of T cell populations

Buffy coats and leukapheresis products were obtained from adult healthy volunteers with approval by the ethical committee (Landesaerztekammer Rheinland-Pfalz und Baden-Wuertemberg). A written informed consent was obtained from all participants.

Human naturally occurring CD4^+^CD25^+^ regulatory T cells (nTregs) and untouched human CD4^+^ T helper cells (Th) were isolated as previously described [13]. As a quality control, purified nTreg cells were stained with CD4, CD25, CD8, CD19, CD16/56 and CD14 (BD and analysed by FACS Canto II (BD Bioscience) (figure S1). Polyclonal T cell activation was performed using soluble 1 µg/ml anti CD3 (clone OKT3) antibody (ebioscience) and 2 µg/ml anti CD28 (clone 28.2) antibody (ebioscience). After 24 h, cultivation in X-Vivo-15 (Lonza), cells were stained for activation markers (figure S2). Mouse nTregs and mouse CD4^+^ Th cells were isolated as described previously by Bopp et al. [28]. All mice used for this study were bred and housed in a specifi c pathogen-free colony at the animal facility of Johannes Gutenberg University using institutionally approved protocols (permission was obtained from the Landesuntersuchungsamt Koblenz, Germany).

### Transfection of primary T cells

The transfection of primary T cells was performed according to the instructions of the manufacturer (AMAXA). Naturally occurring CD4^+^ Th cells (4×10^6^ CD4+ cells/cuvette) were transfected with 1 µM pre miR-155/BIC (Ambion), 1 µM of the mature mimic miR-155 (Dharmacon) respective 2 µM miR-155 inhibitor miRNA (Dharmacon). To determine the unspecific effect of the nucleofection, cells were pulsed without any oligonucleotide (‘pulse only’ control) or with 1 µM control miRNA (Dharmacon). To recover, cells were cultured for additional 24 h after transfection before they were labelled with CFSE [29]. Remaining dead cells were depleted according to the manufacturer's instructions using the Dead Cell Removal kit (Miltenyi Biotec).

### Polyclonal proliferation assay

Transfected and CFSE labelled CD4^+^ Th cells (responder cells) were cultured in 96 well round bottom plates (Nunc) at 1×10^5^ cells/well in the presence or absence of different numbers of freshly isolated allogenic CD4^+^CD25^+^ nTreg cells. To stimulate, cells were treated with 0,5 µg/ml anti CD3 (clone OKT3) and co-cultivated with 1×10^5^ allogenic, antigen presenting cells (APCs). The latter ones were depleted of T cells using CD3 DynaBeads (Invitrogen). After four days of incubation the remaining fluorescence of the CFSE labelled responder cells was analyzed by FACS CantoII (BD Bioscience). The 3H thymidine incorporation assays used for the mouse experiments were performed as described previously [28].

### RNA isolation & quantitative PCR

Total RNA (including miRNAs) was isolated using the mirVana miRNA Isolation Kit (Ambion) according to the manufacturer's instruction and concentration of total RNA was measured by NanoDrop. After transcription into cDNA using the Taqman MicroRNA Reverse Transcription Kit (Applied Biosystems), the expression of miR-155 respective of the normalizer U18 (U5 for mouse miR-155) was quantified with the hsa-miR-155 assay (Applied Biosystems). For the detection of BIC and the corresponding normalizer RNA Pol II, total RNA were converted into cDNA using the High Capacity cDNA Reverse Transcription Kit (Applied Biosystem) and quantified with the assay-on-demand Hs01374569_m1 (Applied Biosystems). Expression levels were calculated as relative fold changes compared to the normalizer and substracted from the normalizer sample (resting CD4^+^ Th cells) based upon the delta-delta CT method.

### Affymetrix Exon Array Profiling

Total RNA were extracted by using RNeasy Plus Mini kits (Qiagen). RNA quality was assessed by using the RNA 6000 Nano Assay (Agilent Technologies). RNA samples were further purified and prepared according to the manufacturer's protocol by using Affymetrix's GeneChip Whole Transcript Sense Target Labeling Assay designed for the Human Exon arrays. Arrays were scanned using the Affymetrix GCS 3000 7G and Gene-Chip Operating Software v. 1.3 to produce. CEL intensity files. Probe-signal intensities were sketch-normalized by using a subset of the ‘extended’ probe sets. Transcript cluster expression was summarized by using a robust multiarray average method (RMA) with a core set of well annotated exons using the ArrayAssist software (Agilent). MIAME-compliant annotated raw data (.cel files) could be found under ArrayExpress entry: E-TABM-779.

### FoxP3 Chromatin-immunoprecipitation (ChIP)

Genpathway's FactorPath method was carried out as described by Labhart et. al (2005) [30]. In brief, human T cell populations, activated for 16 h, were fixed with 1% formaldehyde for 15 min and quenched with 0.125 M glycine. Chromatin was isolated by adding lysis buffer, followed by disruption with a Dounce homogenizer (cells). Lysates were sonicated (Misonix) to shear the DNA to an average length of 300–500 bp. Genomic DNA (Input) was purified from an aliquot of chromatin and quantified on a Nanodrop spectrophotometer. Extrapolation to the original chromatin volume allowed quantitation of the total chromatin yield.

ChIP assays of actived Th cells and nTregs were carried out in duplicate. An aliquot of chromatin (50 ug) was precleared with protein G agarose beads (Invitrogen). FoxP3-bound genomic DNA regions were isolated using an goat polyclonal antibody against FoxP3 (Abcam ab2481). After incubation at 4 C overnight, protein G agarose beads were used to isolate the immune complexes. Complexes were washed, eluted from the beads with SDS buffer, and subjected to RNase and proteinase K treatment. Crosslinks were reversed by incubation overnight at 65 C, and ChIP DNA was purified by phenol-chloroform extraction and ethanol precipitation. To assay for the enrichment of positive control region (PDE3a & IL7R – data not shown) in the ChIP DNA, quantitative PCR (QPCR) reactions were carried out in triplicate with primers specific for these regions using SYBR Green Supermix (Bio-Rad). The resulting signals were normalized for primer efficiency by carrying out QPCR for each primer pair using Input DNA (data not shown).

### Illumina Sequencing

Remaining ChIP DNA (90% of entire sample) was amplified using the Illumina ChIP-Seq DNA Sample Prep Kit. In brief, DNA ends were polished and 5′-phosphorylated using T4 DNA polymerase, Klenow polymerase and T4 polynucleotide kinase. After addition of 3′-A to the ends using Klenow fragment (3′-5′ exo minus), Illumina genomic adapters were ligated and the sample was size-fractionated (∼175–225 bp) on a 2% agarose gel. After a final PCR amplification step (18 cycles, Phusion polymerase), the resulting DNA libraries were quantified and tested by QPCR at the same specific genomic regions as the original ChIP DNA to assess quality of the amplification reactions. DNA libraries were sent to Illumina Sequencing Services for sequencing on a Genome Analyzer II. Sequences (35 bases; ∼14 million quality-filtered sequences/sample) were aligned to the human genome (NCBI Build 36.3) using Eland software. Aligns were extended in silico at their 3′-ends to a length of 110 bp, which is the average genomic fragment length in the size selected library, and assigned to 32-nt bins along the genome. The resulting histograms were stored in BAR (Binary Analysis Results) files. Peak locations were determined by applying a threshold of 18 (5 consecutive bins containing >18 aligns) and storing the resulting intervals in BED files (Affymetrix TAS software). These files were analyzed using Genpathway proprietary software that provides comprehensive information on genomic annotation, peak metrics and sample comparisons for all peaks (intervals). In addition the BED files were used to upload to the UCSC Genome Browser and/or Integrated Genome Browser (Affymetrix) to generate the captures of bound genomics regions surrounding annotated miRNA loci.

## Supporting Information

Figure S1 Purification control of CD4+ Th cells and CD4+CD25+ nTreg cells by FACS analysis. CD4+ Th cells and nTregs were isolated from human leukapheresis (up to 1.5×1010 whole PBMC cells) of healthy volunteers. Populations were stained with the indicated markers using fluorescence-labeled mAbs and analyzed by flow cytometry. The figure shows the dot blot analysis of ungated cell measurement. The cells were stained with CD4 APC (BD Biosciences), CD25 PE-Cy5 (BD Bioscience), CD25 PE-Cy7 (BD Biosciences), CD8 PerCp-Cy5.5 (BD Biosciences) and CD19 APC-Cy7 (BD Biosciences), CD3 FITC/CD56/CD16 PE (BD Bioscience) or CD14 FITC (BD Biosciences) and analysed by FACS CantoII (BD Bioscience). The purity of the resulted nTregs was typically >94% and for CD4+ Th cells >98%.
(0.89 MB TIF)

Figure S2 Isolated human T cell populations conduct normal for activation markers. The activation control of the nTreg and CD4+ Th cells was tested by a polyclonal T cell activation using soluble 1 µg/ml anti CD3 (clone OKT3) antibody (ebioscience) and 2 µg/ml anti CD28 (clone 28.2) antibody (ebioscience). The cells were cultured in Xvivo-15 medium (Cambrex) for 24 h, then the activation level was FACS analysed by detecting specific Treg- and activation markers like: FoxP3, CD127, CD25, CTLA4, GITR and the adhesion marker ICAM-1.
(2.50 MB TIF)

Figure S3 In silico identification of putative miR-155 mRNA targets in CD4+ Th cells. An in silico predicted miR-155 target gene list was generated by analysis of available microRNA databases (miRanda, PicTar, TargetScanS). A comparison of the complete list of 1774 predicted targets to the expression levels generated out the Affymetrix Exon Array profiling was performed. Out of 1774 transcript a list of 867 non-redundant transcripts were found to be represented on the Affymetrix Exon arrays. 321 genes were not expressed in human T cells populations, neither in CD4+ Th and nTreg cells, nor under resting or stimulated conditions. The remaining 546 predicted miR-155 targets expressed in T cells were divided in regulated genes upon miR-155 expression (300 genes) and genes which showed no regulation upon stimulation (246 genes). 199 genes out of these 300 regulated miR-155 targets were excluded showing an up-regulation after T cell activation. Finally 93 predicted miR-155 target genes were left which displayed a down-regulation after T cell activation and are listed as miR-155 targets (supporting table 2).
(5.67 MB TIF)

Figure S4 Predicted miR-155 target genes display down-regulated mRNA expression after cell activation. mRNA of an time course dependent activation of human CD4+ Th cells were analysed for human miR-155 target gene expression using Taqman RT-PCR: The fold changes of the following selected miR-155 predicted target genes are shown: (A) transcription factor HIVEP2; (B) transcription factor BTB and CNC homology 1 (Bach1); (C) transcriptional repressors MXI1; (D) small Maf family protein K (MafK); (E) repressor Transducin-like enhancer protein 4 (TLE4); (F) transcriptional repressor HMG box transcription factor 1 (HBP1). All putative miR-155 targets illustrate the strongest down-regulation between 16 h and 24 h after stimulation with anti-CD3 and anti-CD28 antibodies. All values were calculated and shown as relative fold changes using the ddCT method. As normalizer human RNA Pol II was used.
(7.19 MB TIF)

Table S1 FoxP3-bound genomic loci with annotated micro-RNAs. Listed are the bound micro-RNAs, the underlying chromosome, the significance of positive binding in the appropriate T cell population (-: no significant binding at all; +: one out of two donors showed significant FoxP3 binding; ++: both donors showed FoxP3 binding, the regions were nearby located, but still not overlapping; +++: both donors revealed significant overlapping FoxP3-binding), the category of localization of the miRNA (intra-, intergenic & promotor), associated gene(s) and genomic view captures indicating the bound region (as a pure box & as miniaturized bar graphs).
(0.62 MB PDF)

Table S2 List of T lymphocyte-specific miR-155 target genes. Listed are 93 genes with their gene symbol, gene description, RefSeq identifier, Ensemble transcript number, the relative expression values of the Affymetrix Exon arrays in log2 values (average of 10 human donors) of the six profiled population of T cells (Fig. 1A), the fold changes (as log2 ratios) of 16 h activated vs. non-activated CD4+ Th cells, the significance of the fold change of 16 h activated vs. non-activated CD4+ Th cells as negative log10 p-values generated using the paired Student T-test, the fold changes (as log2 ratios) of 16 h activated vs. non-activated nTregs & the significance of the fold change of 16 h activated vs. non-activated nTregs as negative log10 p-value generated using the paired Student T-test. The heat map-like color code shows the expression level gradient from highly expressed target genes (red) over moderate expressed (orange) to very low expressed candidates (green). The arrow in the fold change columns symbolizes the moderate (yellow) to strong down-regulation (red) upon activation of the T cell populations. The circle in the significance columns is categorizing the p values to be significant (p-val <0.01; green) or not (red).
(1.14 MB EPS)
