# Supplementary figures and images for: miR-155 Inhibition Sensitizes CD4+ Th Cells for TREG Mediated Suppression
Source: PLoS One. 2009 Sep 24;4(9):e7158. doi: 10.1371/journal.pone.0007158 (PMC2743997; doi:10.1371/journal.pone.0007158)

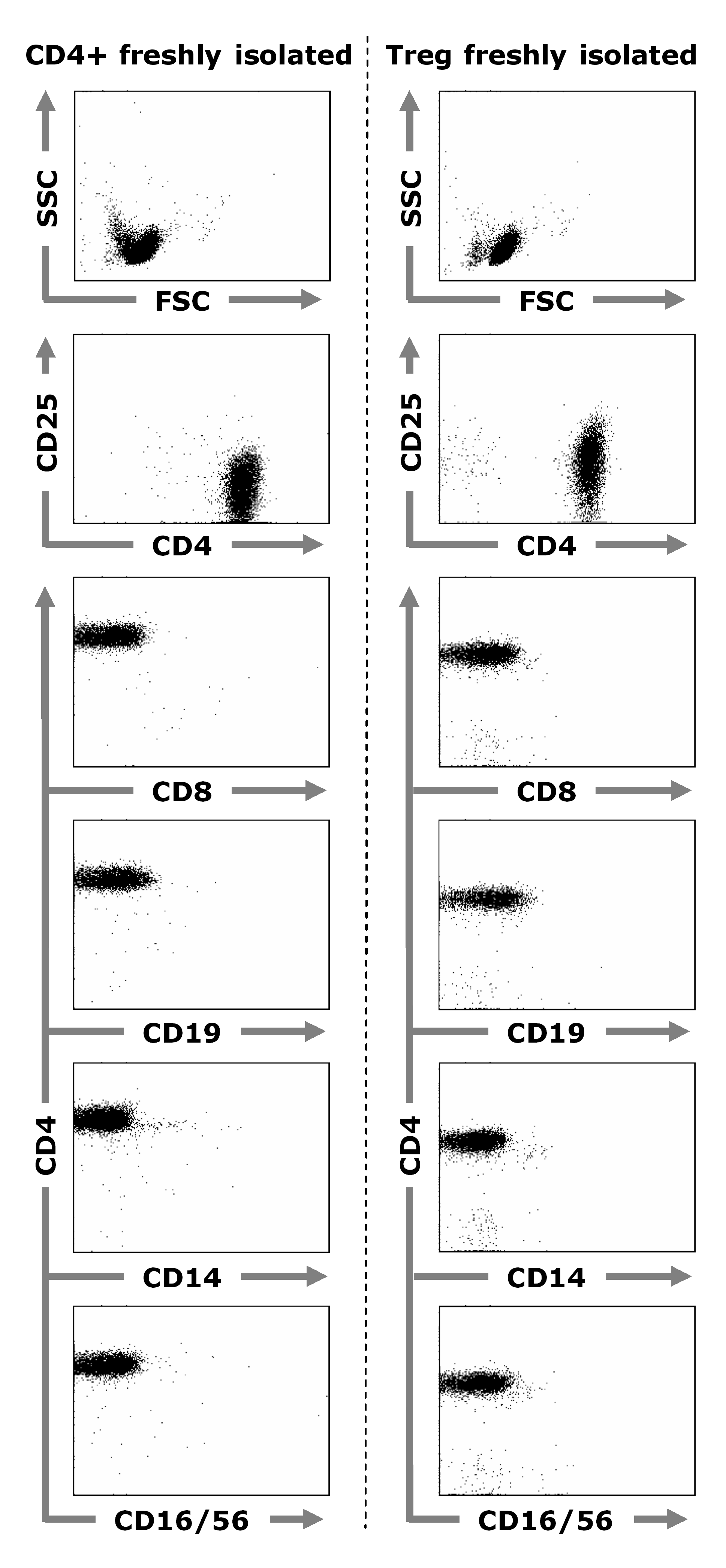

Supplement: Figure S1 — Purification control of CD4+ Th cells and CD4+CD25+ nTreg cells by FACS analysis. CD4+ Th cells and nTregs were isolated from human leukapheresis (up to 1.5×10^10^ whole PBMC cells) of healthy volunteers. Populations were stained with the indicated markers using fluorescence-labeled mAbs and analyzed by flow cytometry. The figure shows the dot blot analysis of ungated cell measurement. The cells were stained with CD4 APC (BD Biosciences), CD25 PE-Cy5 (BD Bioscience), CD25 PE-Cy7 (BD Biosciences), CD8 PerCp-Cy5.5 (BD Biosciences) and CD19 APC-Cy7 (BD Biosciences), CD3 FITC/CD56/CD16 PE (BD Bioscience) or CD14 FITC (BD Biosciences) and analysed by FACS CantoII (BD Bioscience). The purity of the resulted nTregs was typically >94% and for CD4+ Th cells >98%. (0.89 MB TIF) [file pone.0007158.s001.tif]

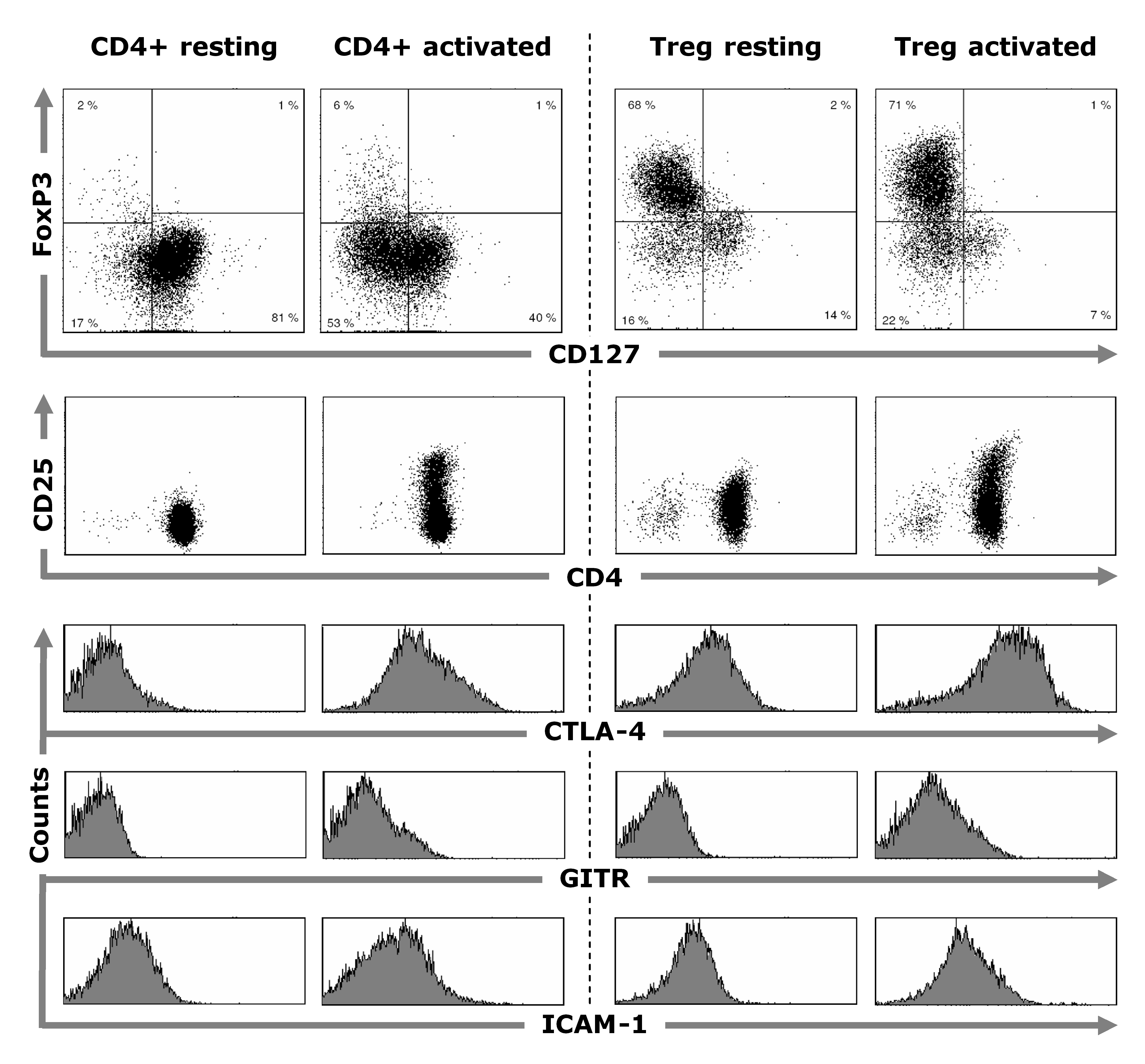

Supplement: Figure S2 — Isolated human T cell populations conduct normal for activation markers. The activation control of the nTreg and CD4+ Th cells was tested by a polyclonal T cell activation using soluble 1 µg/ml anti CD3 (clone OKT3) antibody (ebioscience) and 2 µg/ml anti CD28 (clone 28.2) antibody (ebioscience). The cells were cultured in Xvivo-15 medium (Cambrex) for 24 h, then the activation level was FACS analysed by detecting specific Treg- and activation markers like: FoxP3, CD127, CD25, CTLA4, GITR and the adhesion marker ICAM-1. (2.50 MB TIF) [file pone.0007158.s002.tif]

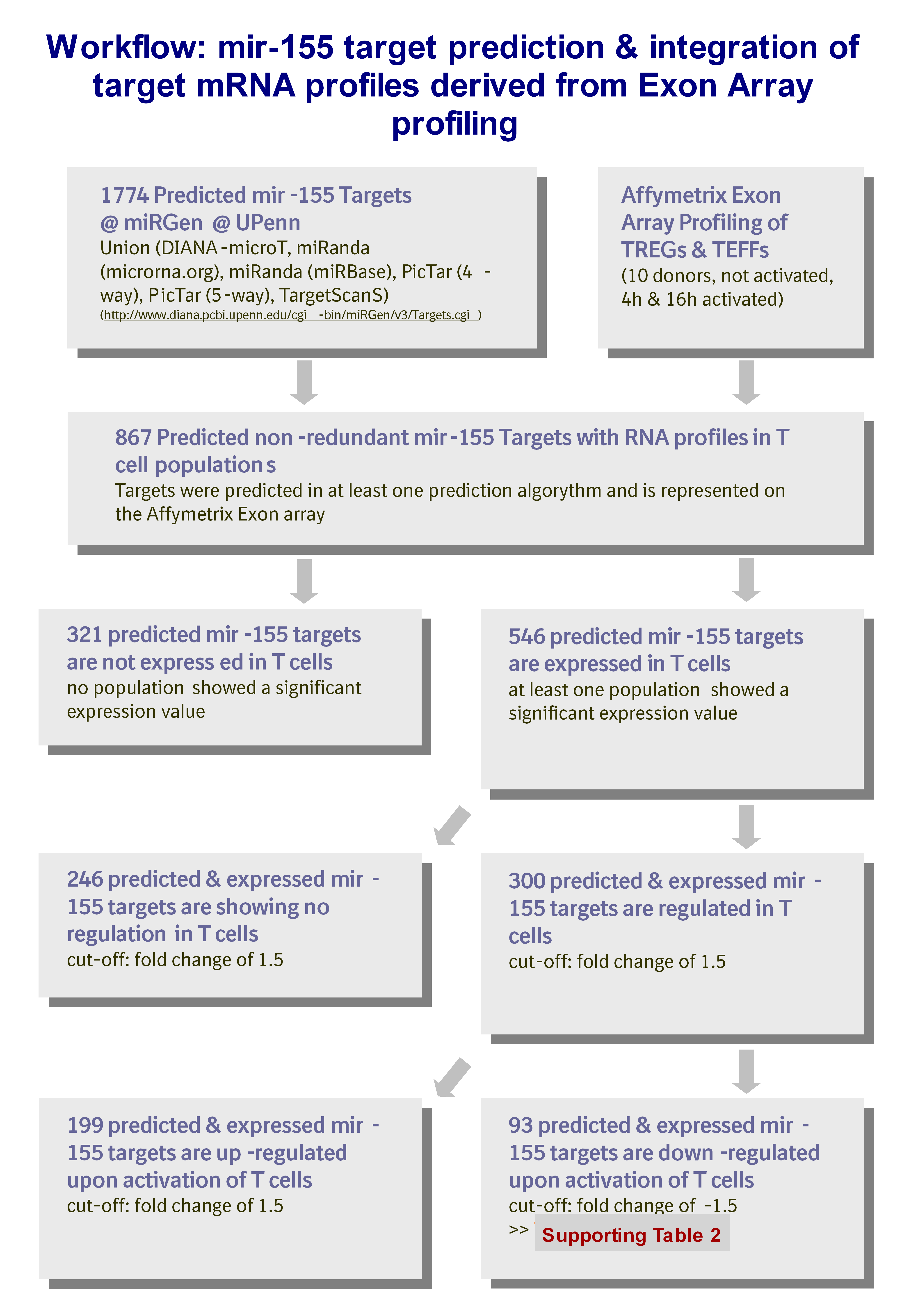

Supplement: Figure S3 — In silico identification of putative miR-155 mRNA targets in CD4+ Th cells. An in silico predicted miR-155 target gene list was generated by analysis of available microRNA databases (miRanda, PicTar, TargetScanS). A comparison of the complete list of 1774 predicted targets to the expression levels generated out the Affymetrix Exon Array profiling was performed. Out of 1774 transcript a list of 867 non-redundant transcripts were found to be represented on the Affymetrix Exon arrays. 321 genes were not expressed in human T cells populations, neither in CD4+ Th and nTreg cells, nor under resting or stimulated conditions. The remaining 546 predicted miR-155 targets expressed in T cells were divided in regulated genes upon miR-155 expression (300 genes) and genes which showed no regulation upon stimulation (246 genes). 199 genes out of these 300 regulated miR-155 targets were excluded showing an up-regulation after T cell activation. Finally 93 predicted miR-155 target genes were left which displayed a down-regulation after T cell activation and are listed as miR-155 targets (supporting table 2). (5.67 MB TIF) [file pone.0007158.s003.tif]

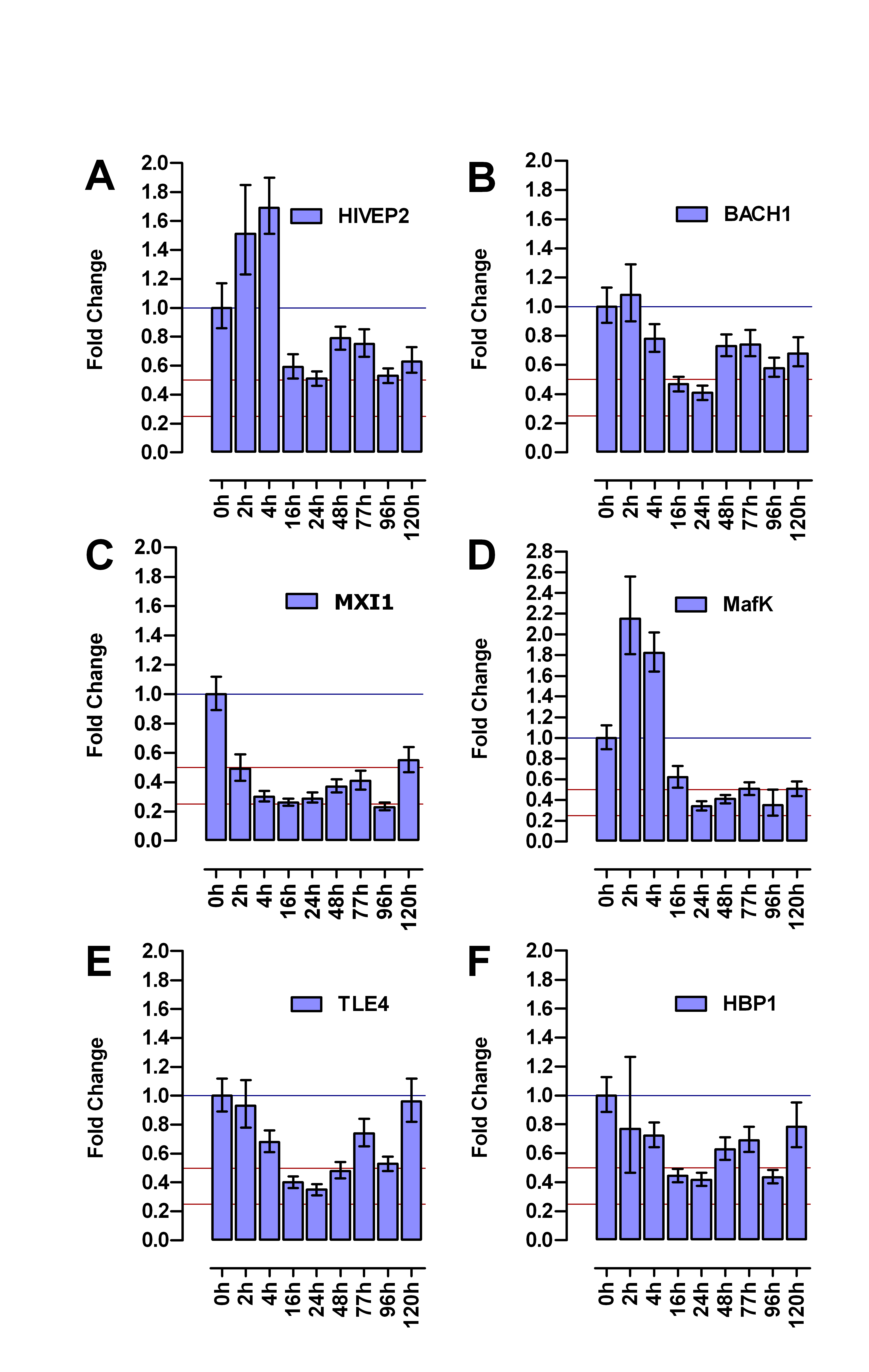

Supplement: Figure S4 — Predicted miR-155 target genes display down-regulated mRNA expression after cell activation. mRNA of an time course dependent activation of human CD4+ Th cells were analysed for human miR-155 target gene expression using Taqman RT-PCR: The fold changes of the following selected miR-155 predicted target genes are shown: (A) transcription factor HIVEP2; (B) transcription factor BTB and CNC homology 1 (Bach1); (C) transcriptional repressors MXI1; (D) small Maf family protein K (MafK); (E) repressor Transducin-like enhancer protein 4 (TLE4); (F) transcriptional repressor HMG box transcription factor 1 (HBP1). All putative miR-155 targets illustrate the strongest down-regulation between 16 h and 24 h after stimulation with anti-CD3 and anti-CD28 antibodies. All values were calculated and shown as relative fold changes using the ddCT method. As normalizer human RNA Pol II was used. (7.19 MB TIF) [file pone.0007158.s004.tif]
