# Supplementary material for: miR-155 Inhibition Sensitizes CD4+ Th Cells for TREG Mediated Suppression
Source: PLoS One. 2009 Sep 24;4(9):e7158. doi: 10.1371/journal.pone.0007158 (PMC2743997; doi:10.1371/journal.pone.0007158)
Supplement: Table S1 — FoxP3-bound genomic loci with annotated micro-RNAs. Listed are the bound micro-RNAs, the underlying chromosome, the significance of positive binding in the appropriate T cell population (-: no significant binding at all; +: one out of two donors showed significant FoxP3 binding; ++: both donors showed FoxP3 binding, the regions were nearby located, but still not overlapping; +++: both donors revealed significant overlapping FoxP3-binding), the category of localization of the miRNA (intra-, intergenic & promotor), associated gene(s) and genomic view captures indicating the bound region (as a pure box & as miniaturized bar graphs). (0.62 MB PDF) [file pone.0007158.s005.pdf]

Supporting Table 1: FoxP3-bound genomic loci with annotated micro-RNAs.

miRNA	Chr.	CD4	CD25	Location	Genes associated	Genomic Vizualization (UCSC Browser)	Genomic Vizualization (UCSC Browser)
mir-1255a	4	+++	+++	intragenec	PPP3CA	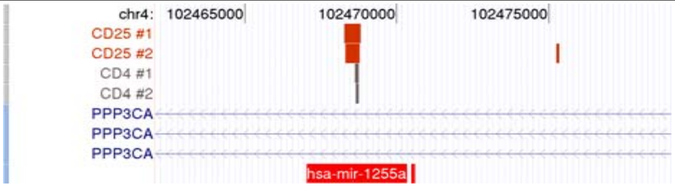	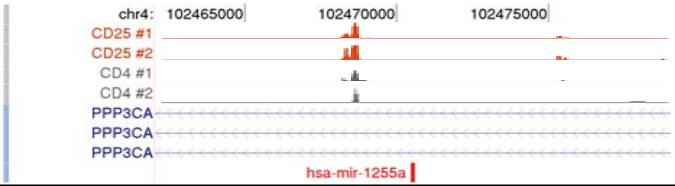
mir-1259	20	+	+++	promotor	ZNFX1, C20ORF199	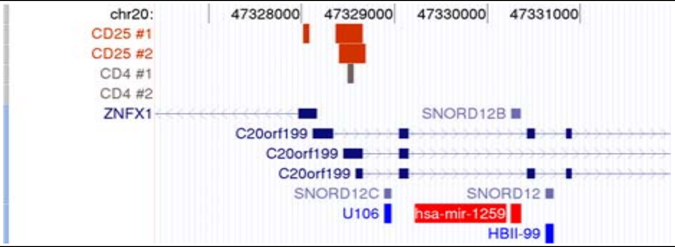	
mir-1268	15	+	+++	intergenic	-	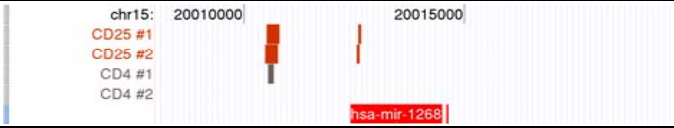	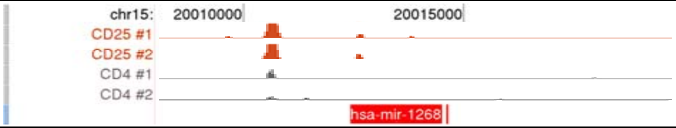
mir-1281	22	+	+++	promotor	EP300	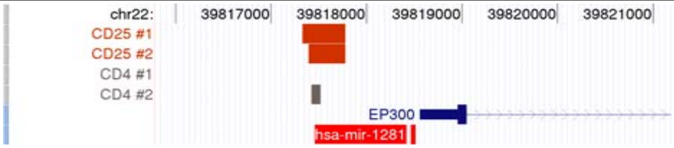	
mir-1292	20	-	+++	promotor	NOP56	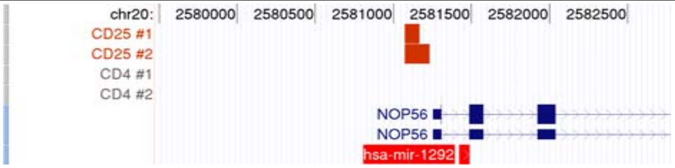	
mir-1302-2	15	+++	+++	promotor	WASH3P	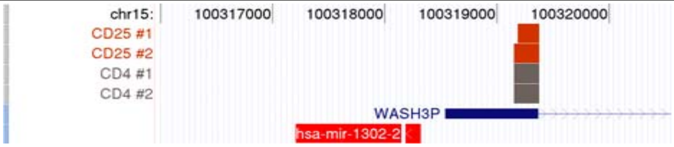	
mir-1302-3	2	+++	+++	promotor	WASH2P	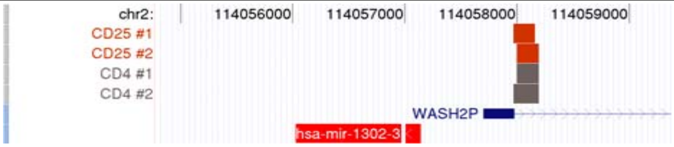	

Supporting Table 1: FoxP3-bound genomic loci with annotated micro-RNAs.

miRNA	Chr.	CD4	CD25	Location	Genes associated	Genomic Vizualization (UCSC Browser)	Genomic Vizualization (UCSC Browser)
mir-138-2	16	-	+++	intergenic	-	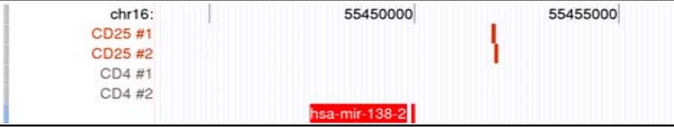	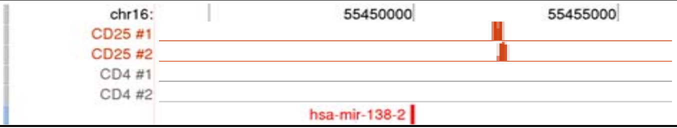
mir-141 mir-200c	12	-	+++	intergenic		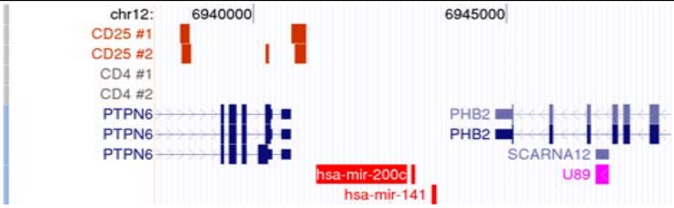	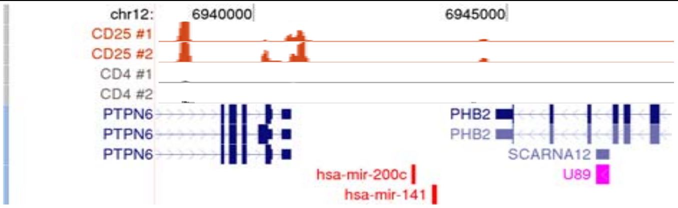
mir-142	17	-	+++	intergenic, promotor	BZRAP1	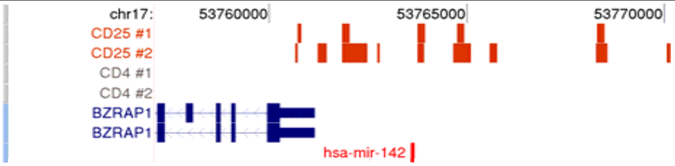	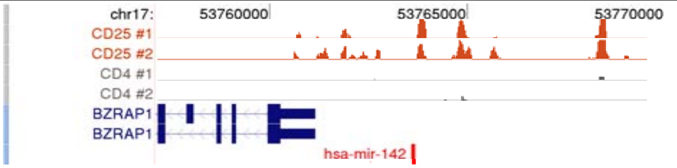
mir-146a	5	-	+++	intragenec		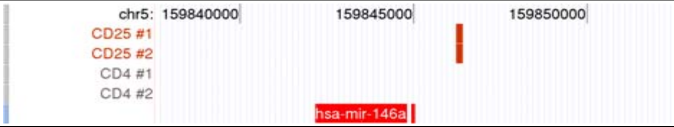	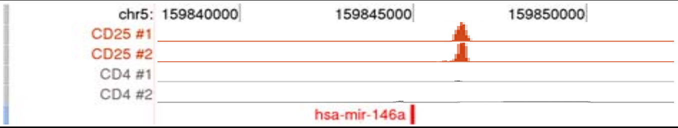
mir-150	19	-	+++	intergenic	-	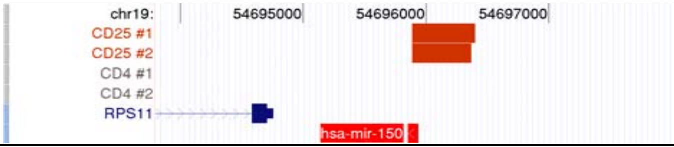	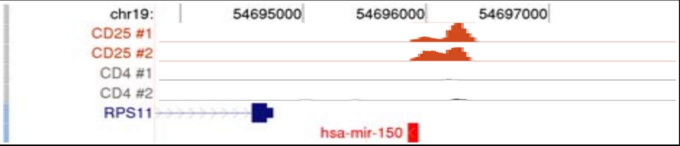
mir-153-1	2	-	+++	intragenec	PTPRN	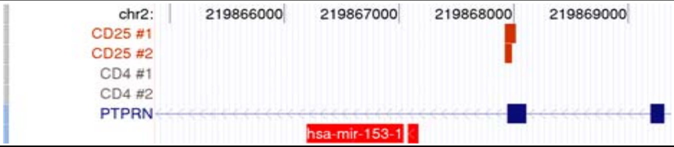	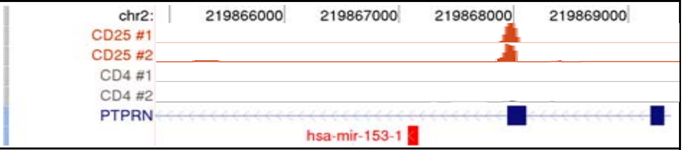
mir-155	21	-	+++	intergenic		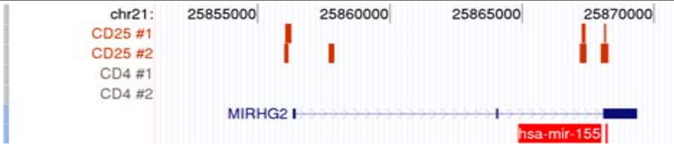	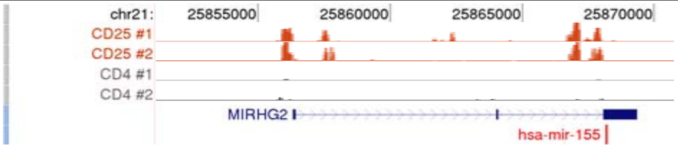

Supporting Table 1: FoxP3-bound genomic loci with annotated micro-RNAs.

miRNA	Chr.	CD4	CD25	Location	Genes associated	Genomic Vizualization (UCSC Browser)	Genomic Vizualization (UCSC Browser)
mir-17 mir-18a mir-19a mir-20a mir-19b-1 mir-92a-1	13	-	+++	intergenic		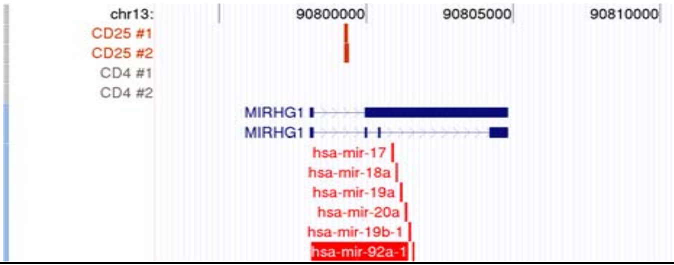	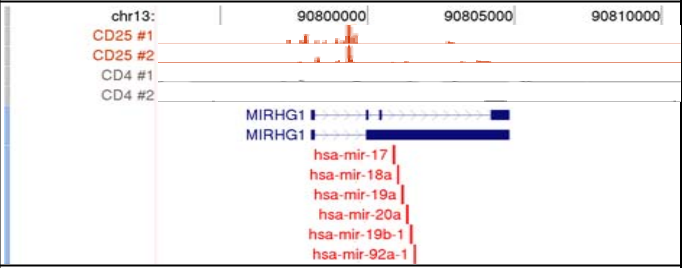
mir-21	17	-	+++	intergenic		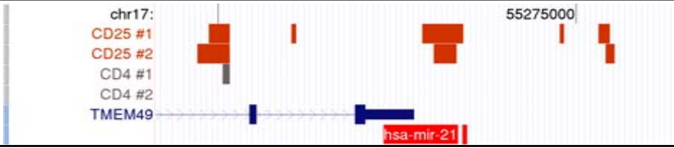	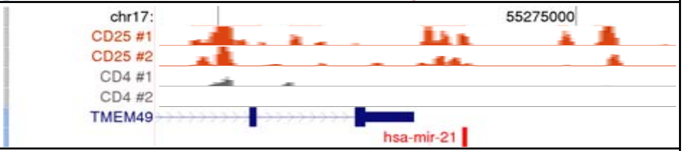
mir-22	17	+	+++	promotor	C17ORF91	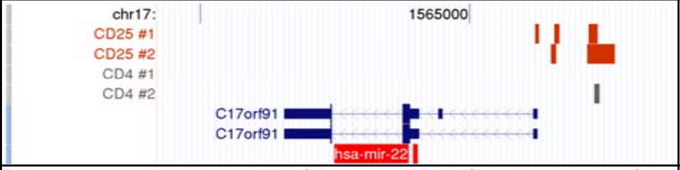	
mir-23a mir-24-2 mir-27a	19	-	+++	intergenic	-	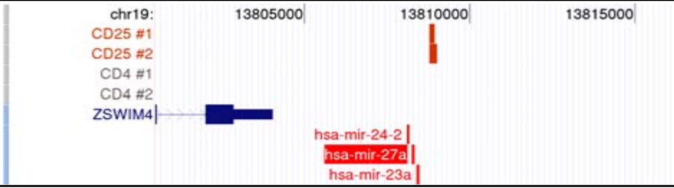	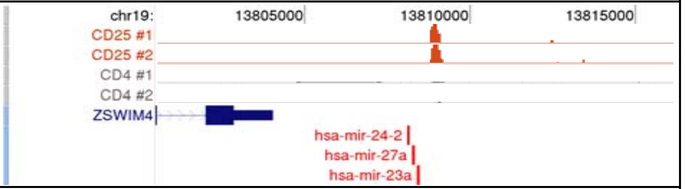
mir-26b	2	+++	+++	promotor	CTDSP1	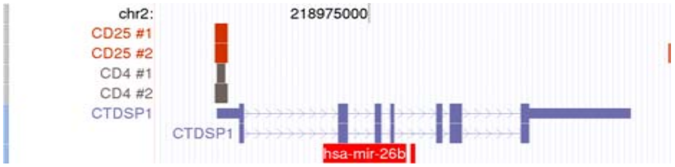	
mir-484	16	-	+++	promotor	KIAA0430 NDE1	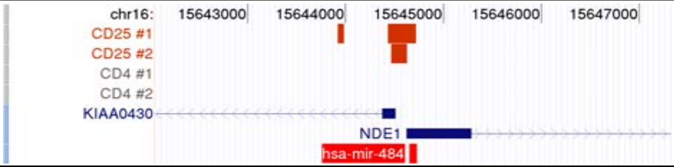	

Supporting Table 1: FoxP3-bound genomic loci with annotated micro-RNAs.

miRNA	Chr.	CD4	CD25	Location	Genes associated	Genomic Vizualization (UCSC Browser)	Genomic Vizualization (UCSC Browser)
mir-568	3	-	+++	intergenic	-	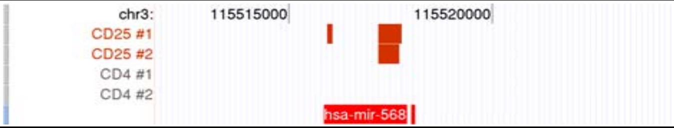	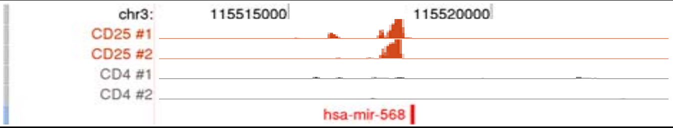
mir-607	10	-	+++	promotor	LCOR	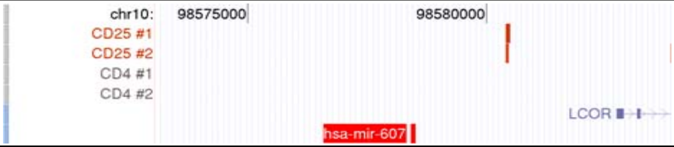	
mir-611	11	-	+++	promotor	FEN1, C11ORF10	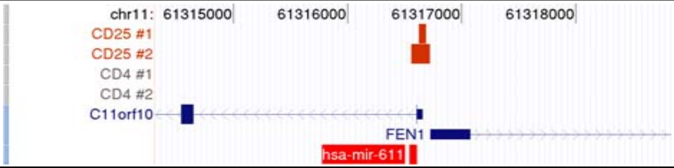	
mir-619	12	-	+++	intragenec	SSH1	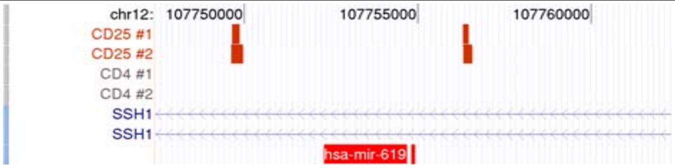	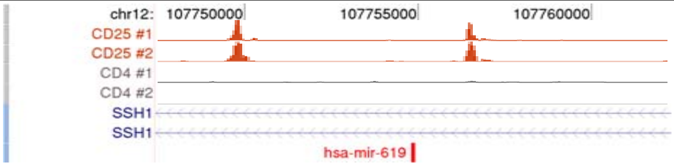
mir-623	13	-	+++	intragenec	UBAC2	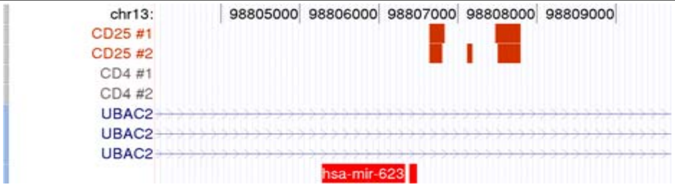	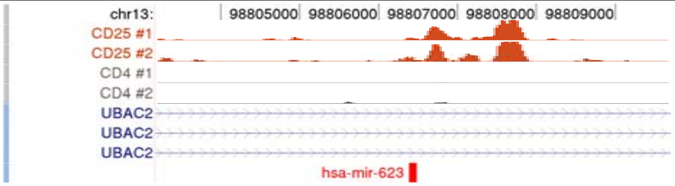
mir-632	17	+	+++	promotor	ZNF207	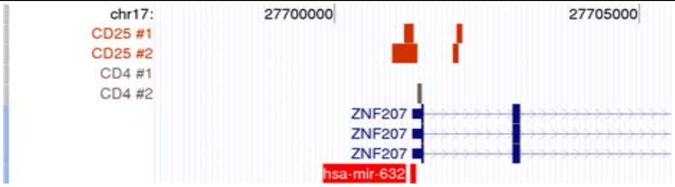	
mir-636	17	-	+++	promotor	SFRS2 MFSD11	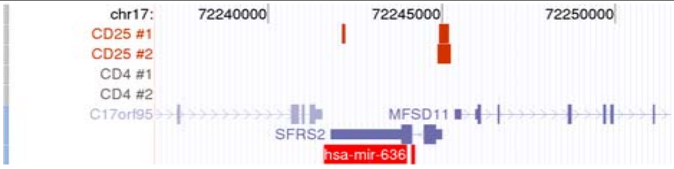	

Supporting Table 1: FoxP3-bound genomic loci with annotated micro-RNAs.

miRNA	Chr.	CD4	CD25	Location	Genes associated	Genomic Vizualization (UCSC Browser)	Genomic Vizualization (UCSC Browser)
mir-638	19	++	+++	promotor	DNM2	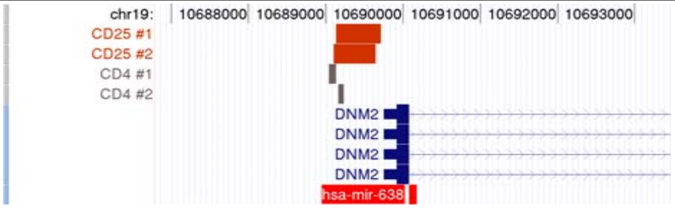	
mir-650	22	-	+++	intergenic	-	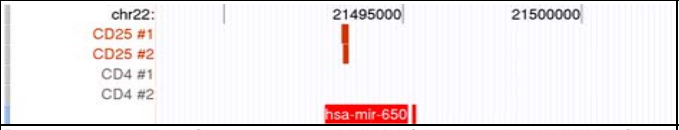	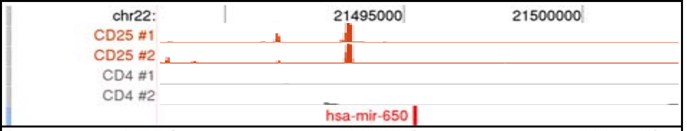
mir-760	1	+	+++	intergenic		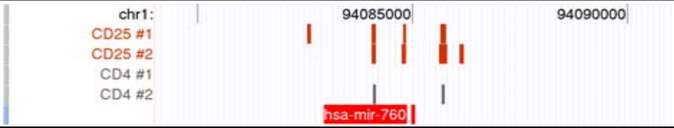	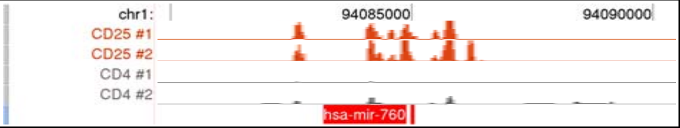
mir-766	X	-	+++	intragenec	SEPTIN6	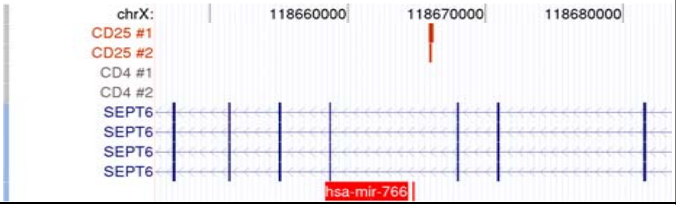	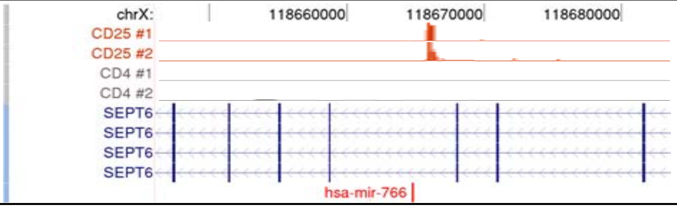
mir-923	17	++	-	intragenec	UNC45B	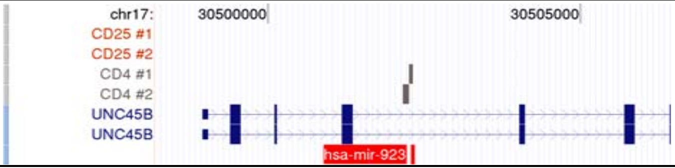	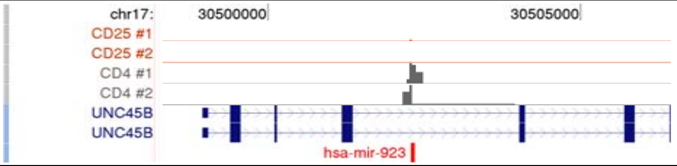
mir-933	2	+	+++	promotor	ATF2	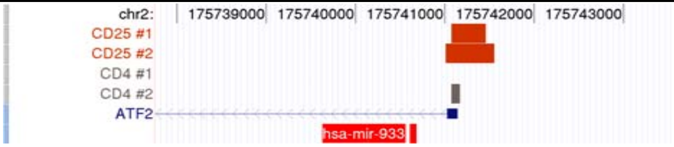	
U8	17	+	+++	3'UTR	TMEM107	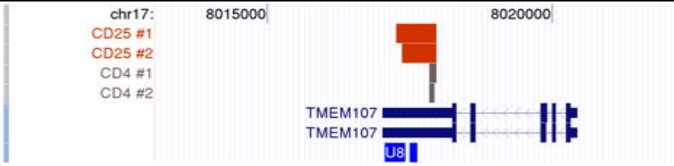	
